# Trade-offs, control conditions, and alternative designs in the experimental study of cultural evolution

**DOI:** 10.1073/pnas.2322886121

**Published:** 2024-11-18

**Authors:** Maxime Derex, Pierce Edmiston, Gary Lupyan, Alex Mesoudi

**Affiliations:** ^a^Institute for Advanced Study in Toulouse, Toulouse School of Economics, Toulouse 31080, France; ^b^Department of Psychology, University of Wisconsin-Madison, Madison, WI 53706; ^c^Centre for Ecology and Conservation, University of Exeter, Cornwall TR10 9FE, United Kingdom

**Keywords:** cultural evolution, cumulative culture, laboratory experiments, social learning

## Abstract

Although the theoretical foundations of the modern field of cultural evolution have been in place for over 50 y, laboratory experiments specifically designed to test cultural evolutionary theory have only existed for the last two decades. Here, we review the main experimental designs used in the field of cultural evolution, as well as major findings related to the generation of cultural variation, content- and model-based biases, cumulative cultural evolution, and nonhuman culture. We then identify methodological advances that demonstrate the iterative improvement of cultural evolution experimental methods. Finally, we focus on one common critique of cultural evolution experiments, the appropriate individual learning control condition needed to demonstrate cumulative culture, and present an original experimental investigation relevant to this critique. Participants completed a combinatorial innovation task allowing for cumulative improvement over time in one of four commonly used experimental designs/conditions: social learners in chains, social learners in groups, individual learners experiencing an extended session lasting the same accumulated time as an entire chain or group, and individual learners experiencing repeated sessions adding up to the same total time. We found that repeated individual learning resulted in superior performance to any other condition. We discuss these findings in light of the relevance of the specific criticism of previous experimental studies that purport to have demonstrated cumulative culture. We also use our findings to discuss the broad trade-offs that participants face when learning individually and socially in different contexts, including variable acquisition costs, redundancy of effort in groups, and cognitive and motivational fatigue.

## Significance

In the last two decades, laboratory experiments have been used to test the assumptions and predictions of cultural evolutionary theory as developed over the last 50 y. Here, we review commonly used experimental designs, key findings, and methodological advances relating to cultural evolution experiments. We present an original experimental investigation that compares commonly used experimental designs (chains and groups) and control conditions (extended and repeated individual learning), and in so doing explore the various trade-offs that participants face when engaging in individual and social learning in different contexts.

Fifty years ago, Cavalli-Sforza and Feldman (1, 2) laid the theoretical foundations for the modern field of cultural evolution, in which cultural change is viewed as an evolutionary process (3–5). In these and subsequent works (6), Cavalli-Sforza and Feldman borrowed and adapted the theoretical tools of population genetics to formalize the processes that comprise and shape cultural evolution: cultural traits are transmitted via various pathways (e.g. vertical, oblique or horizontal), are subject to cultural selection where some traits are more likely to be learned and passed on than others, and new cultural variation emerges via cultural mutation and migration.

The first explicit empirical tests of this body of theory were ethnographic studies mapping the pathways of transmission in small-scale communities (7). Subsequent key developments included the comparative analysis of cross-cultural variation in languages and behavioral practices (8, 9) and studies of nonhuman cultural transmission and variation (10, 11). It was not until the early 2000s that lab experiments (12–15) were used to explicitly test the assumptions and predictions of the cultural evolutionary theory laid out by Cavalli-Sforza and Feldman and others.

Experiments provide an important link between theoretical models and analyses of historical, linguistic, ethnographic, and other real-world data (16). Experiments maintain some of the control and simplicity of theoretical models, allowing us to manipulate or control variables, randomly assign participants to different conditions, and directly observe behavior, features which are seldom possible for observational or historical methods. Yet unlike models, experiments involve actual human or nonhuman behavior and cognition, rather than a modeler’s intuitions about how individuals behave and think. While experiments necessarily maintain a degree of artificiality and simplicity compared to the real world, when used in conjunction with theoretical models and real-world data, they provide a key element of a broad science of cultural evolution (17), just as experiments have played a vital role in evolutionary biology, from Darwin, Mendel and Morgan to modern experimental studies of microorganism evolution (18).

Central to any evolutionary process is inheritance, which in cultural evolution takes the form of social learning. Hence, experimental studies of cultural evolution typically involve participants learning from at least one other participant. Several specific designs have emerged (16). Transmission chain designs involve a written text, task solution, or manufactured artifact being passed along a linear chain of participants, with each participant able to read or see the previous participant’s output. Group-based designs involve groups of participants repeatedly making choices or attempting to solve problems, with participants permitted to see other group members’ choices or solutions. Sometimes group membership is fixed, sometimes group members are periodically replaced, the latter simulating population turnover or migration.

## Key Findings

Key findings from the last two decades of cultural evolution experiments include the following:

### Cultural Mutation.

Early cultural evolution models made the simplifying assumption that new cultural variation emerges via random mutation, analogous to random genetic mutation (6). Subsequent work added greater psychological realism to these models. For example, the accumulated copying error (ACE) model (19, 20) assumes a chain of artifact manufacturers (e.g. handaxe knappers) each of whom tries to exactly copy the size of the artifact produced by the previous individual in the chain. Evidence from psychophysics suggests that size differences of less than 3% (the “Weber fraction”) are imperceptible to human visual systems. Hence the ACE model assumes that each person in the chain produces an artifact that deviates randomly in size somewhere between ±3% of the previous artifact. Across multiple independent chains, this should result in no change in the mean size of the artifact and an increase in the variance, as artifacts in some chains randomly get larger and other chains randomly smaller. To empirically test this model, Kempe et al. (21) had participants in multiple independent chains resize handaxe images to match as accurately as possible the size of the previous participant’s handaxe. As predicted, the variance in handaxe size increased over time consistent with a Weber fraction of 3.43%, similar to the previously assumed value. A subsequent study went beyond simply resizing images of artifacts and had participants in chains actually make clay figurines (23). Here, the Weber fraction was ± 8% and ± 15% for experts and nonexperts respectively. These larger values likely incorporate the added copying error introduced via the manufacturing process, beyond simply errors in perception. Moreover, the smaller copying error for experts indicates important individual differences in cultural mutation due to experience, rather than the models’ assumption of human universality.

### Content Biases.

One form of cultural selection (6) entails certain kinds of information being more likely to be remembered and transmitted than other kinds, often known as content biases (3). Building on classic social psychological methods (22), transmission chain studies have used written stories to reveal content biases that favor the transmission of negative over positive information (23, 24), social over nonsocial information (25, 26), and stereotype-consistent over stereotype-inconsistent information (27). Interestingly, many of these biases are also found when large language models (LLMs) are asked to summarize the same material, rather than actual human participants (28). Given that LLMs are trained on human-generated material from the internet, this suggests that the content biases observed in the confines of the laboratory are also evident in real-world human culture. A similar line of studies has examined the cultural transmission of languages (29) and cognitive representations (30), showing that content (or inductive) biases gradually alter random representations to reflect structural regularities favored by human cognition.

### Model-Based Biases.

In contrast to content biases, model-based biases describe cultural change in response to who is copied, rather than what. Group-based experiments have shown that participants engage in conformity, the disproportionate copying of the most common cultural traits exhibited in the group (31–33), albeit not all participants (34) and not as much as they should in order to maximize payoffs (31). More recent studies have found that conformity is primarily used in response to spatial environmental variation, i.e., when participants move to a new group containing others who have already acquired locally adaptive behavior, rather than temporal environmental variation, i.e., when everyone experiences an environmental shift such that the majority cultural trait is unlikely to yet be adaptive (35), as predicted by theoretical models (36). Other experiments have explored prestige bias, the preferential copying of high-status individuals who are frequently copied and deferred to by others. As predicted, participants engage in prestige bias only when prestige constitutes a reliable cue of demonstrator success rather than a random cue unrelated to payoffs, and only when direct success information about demonstrators is unavailable (37). Furthermore, participants are sensitive to the domain-specificity of prestige cues, preferring to learn from demonstrators who acquired prestige in the same knowledge domain as the trait being copied (38).

### Cumulative Culture.

Much attention has focused on why and how human culture is distinctively cumulative, in the sense that beneficial modifications are preserved, recombined, and accumulated over successive generations to generate technologies and practices that could not have been created by a single individual alone (39). Several studies have sought to simulate cumulative cultural evolution in the lab, with chains of participants modifying and transmitting artifacts like paper airplanes, hand-axes, and baskets (40–42) or solutions to challenging problems (43, 44), identifying the conditions under which artifact performance or solution effectiveness improves over successive experimental “generations.” Studies have shown that cumulative culture is more likely with higher-fidelity transmission mechanisms such as teaching or language (41, 42), and in larger or partially connected groups (45, 46), supporting model predictions (47, 48). Other studies have shown that cumulative culture can occur in the absence of accurate causal understanding of why the transmitted trait improves performance (43); all that is needed is selective copying of successful solutions (44).

### Nonhuman Culture.

Experiments have played a central role in demonstrating the existence and form of cultural evolution in nonhuman species, particularly in the face of skepticism that culture exists in other species. Transmission chain studies demonstrate that chimpanzees are capable of transmitting task solutions with enough fidelity to maintain persistent cultural traditions (49), supporting the cultural basis of such traditions observed in the wild (11), as well as content biases in birdsong (50). Other studies have revealed specific model-based biases such as conformity in birds (51) and payoff bias in monkeys (52). There is also evidence for some form of cumulative culture in pigeons (53), using identical transmission-replacement methods used in humans (40). While some of these studies use captive animals in the lab, many employ field experiments to study animals in the wild, thus achieving greater ecological validity.

## Methodological Advances

The past two decades have also seen advances in experimental methods used to study cultural evolution. Here, we highlight three such advances.

### Transmission Chain Modifications.

Linear transmission chain designs have been criticized for their lack of realism, given that real-world transmission often involves repeatedly learning from multiple demonstrators, rather than a single learning episode from the single preceding individual in the chain (54). While undoubtedly true, the value of experiments, like models, often lies in their simplicity; abstracting away from all other real-world complexities is often necessary to better understand the effect of specific variables. Nevertheless, transmission chain designs have been adapted to explore the generality of existing findings. For example, one study explicitly compared learning twice from the same demonstrator versus learning once each from two different demonstrators (55), finding better transmission in the latter case. Other studies have probed at which stage in the transmission process content biases operate: the encoding/attention phase, the memory/recall phase, or the choice-to-transmit phase (56). These are valuable extensions of early work using only linear, one-to-one transmission chains.

### Generative Statistical Models.

Unlike in psychology, cultural evolution experiments benefit from an established body of formal modeling going back 50 y, as noted above. Some of the earliest cultural evolution experiments took advantage of this formal foundation to directly fit participant data to existing models of cultural evolutionary processes such as conformity (31, 34, 57). Rather than generic statistical models such as linear regression, these generative or theory-driven models (58) can directly estimate each participant’s tendency to, say, conform to a majority, and how this tendency is affected by environmental change. There is much scope for tighter links between formal theory and experimental methods.

### Large-Scale Online Experimentation.

In the last two decades, the internet has made possible experiments of vastly increased scale and complexity compared to traditional face-to-face laboratory experiments. This development is not unique to cultural evolution, but given the field’s focus on social learning within and between groups of individuals and cumulative change over time, online experimentation platforms are particularly useful here. Recent online experiments feature sample sizes in the thousands (44, 59) with participants often dynamically moved across groups depending on performance, features which are difficult or impossible to achieve in the lab.

## Empirical Case Study: Identifying the Appropriate Individual Learning Control Condition in Cultural Evolution Experiments

Notwithstanding the above empirical and methodological advances, there remain important unresolved issues in the experimental study of cultural evolution. One such issue relates to the suitability of individual learning control conditions in experiments that purport to simulate and investigate cumulative cultural evolution in the lab. In the rest of this paper, we examine this critique in more detail, and report an experimental study that compares the performance of individual and social learners across various experimental designs—transmission chains and static groups—that we discussed above. Our aim is not simply to support or reject a particular choice of control condition, but rather to systematically explore the various trade-offs that participants in different learning situations experience. We hope that this will provide a better foundation for future researchers’ choices of experimental designs and the conditions within them, and allow useful reevaluation of past experimental results.

As noted above, cultural evolution experiments have been used to demonstrate and test hypotheses regarding cumulative cultural evolution. One criterion used by experimenters to determine whether the outcome of a transmission chain experiment constitutes cumulative culture is whether gradual improvement has occurred across generations (40, 42, 43). However, this process-focused criterion might seem a relatively low bar for demonstrating cumulative culture. Some have argued that, in addition to demonstrating gradual improvement, cumulative cultural evolution experiments should demonstrate that the solution has improved beyond what an individual could generate on their own (60). Consequently, some experiments have implemented individual learning control conditions comprising lone participants having the same amount of time to complete the same task as participants in the group or chain (61, 62). Experiments that have used this product-focused criterion show that, under some conditions, participants in groups or chains reach performances higher than that of the best individual learner (62). However, this approach has also been criticized for failing to provide individual learners with appropriately comparable learning time as an entire group or chain (63). A proper comparison, it is argued, would include a baseline individual learning condition in which single participants are given the same amount of time as the entire group or chain, e.g. for a transmission chain of 4 participants each given 25 min, individual learners would get 100 min rather than 25 min. This has been implemented in only a handful of experiments (41, 53).

While informative, we do not think that relying on a product-focused criterion is quite so straightforward, nor that an extended individual learning control condition is fully justified. Indeed, the justification for individual learning control conditions is often to test the equivalent claim regarding real-world cultural evolution: that the end product of cumulative culture exceeds what one isolated individual could achieve alone in a single lifetime with no cultural influence (64). Yet learning times in experiments are far shorter than individual lifespans, and such a claim is surely untestable using experimental methods (65).

More substantively, we are not convinced that simply providing individual learners with the same amount of accumulated time as a group or chain is necessarily comparable to that group or chain, for several reasons. First, participants often get better with practice, albeit with decreasing rates of improvement over trials/time, such that individual learners may quickly get more efficient at completing the task than social learners. This may result in relatively more time to refine their solution and more opportunities to reach higher payoffs. Second, there are trade-offs that social learners often face between learning socially and implementing their solution. For instance, in one of the few experiments to implement an extended individual learning condition, social learners had to simultaneously learn how to make an artifact from a demonstrator and produce their own (41). Consequently, social learners had less time than individual learners to build their artifact and could try out fewer alternative designs. Third, social learners experience information loss to a greater extent than individual learners. In most experiments, transmitted solutions represent a subset of what has been tried out by another participant in the chain or group. This means that the loss of important knowledge will systematically occur in social conditions, whereas individual learners’ knowledge about the set of solutions that they have tried out will be limited only by their own memory capacity. Fourth, motivation might reduce over time. Assuming a chain of 10 social learners, individual learners would need to spend 10 times longer solving the task than each social learner. It is hard to believe that participants’ motivation would remain comparable over such varying time periods. Fifth, in the absence of coordinated division of labor, social learners in groups might invest time solving the same problems (“reinventing the wheel”), while individual learners given the same amount of accumulated time will tend to tackle novel problems. This means that, compared to individual learners, social learners in groups will waste time by engaging in redundant work.

**Fig. 1.**
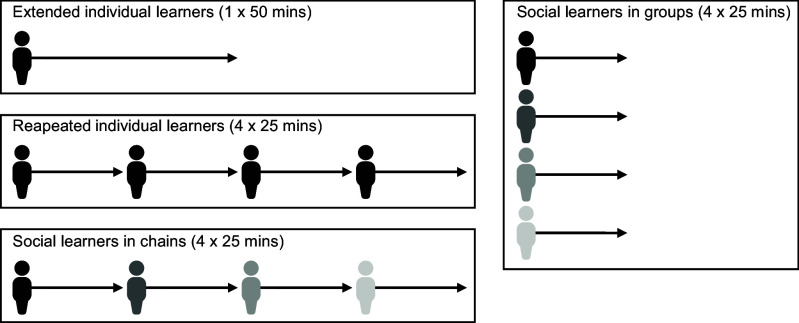
Overview of the experimental conditions. Extended individual learners experienced a single continuous session of 50 min. Repeated individual learners experienced four successive sessions of 25 min on separate days. Social learners experienced a single session of 25 min and were placed in either linear chains of four generations or static groups of either two or four. Repeated individual learners inherited their own innovation record while social learners in chains inherited the record of someone else. Social learners in groups could continuously learn from their other group members.

Given these uncertainties over the appropriateness of individual learning control conditions, we conducted an experimental study that compared the performance of learners across the main experimental designs that are used in the literature on cumulative culture. 570 participants were randomly assigned to one of four experimental designs: individual learners who experienced a single continuous session (hereafter “extended individual learners;” n = 41), individual learners who experienced four successive sessions on separate days (hereafter “repeated individual learners;” n = 59, of which 6 dropped out between sessions), social learners placed in linear chains of four generations (hereafter “chains;” n = 212; 53 distinct chains) and social learners in static groups of either two or four who could continuously learn from other group members (hereafter “groups;” n = 106 or 53 distinct groups of two; and n = 152 or 38 distinct groups of four, respectively). Experimental sessions were 25 min long for all participants except for extended individual learners, who had a single session of 50 min (Fig. 1). The comparison between repeated individual learners and chains allows us to investigate the loss of skill/knowledge and the cost of learning socially. The comparison between chains and groups allows us to investigate the costs of producing redundant work. Finally, the comparison between repeated and extended individual learners allows us to investigate the effect of session duration on individuals’ motivation.

The experiment used the totem task from ref. 62, a combinatorial task in which participants produced virtual “totem poles” (hereafter “totems”). The task simulates real-world innovation in which the production of complex artifacts (here, virtual “totem poles”) depends on the discovery of high-level innovations (e.g., axes), whose discovery is in turn contingent on the discovery of lower-level innovations (e.g., stone tools), with both low- and high-level innovations resulting from a specific production process. Once a tool is discovered, its “recipe,” i.e. the items that must be combined to create it, is displayed in an “innovation record.” Participants can review their own past discoveries by clicking on a tool to see its recipe. Participants’ innovation records also allow the sharing of information within groups and across chains/sessions. Observing an innovation record provides the recipes for creating the tools, but not the tools themselves. To use these tools in further combinations, the tools, including all of the constituent parts, must first be reproduced by the participant observing the innovation record.

Repeated individual learners and social learners in chains experienced the same conditions, each starting a new session by inheriting the innovation record produced during the previous session. The only difference is that repeated individual learners inherited their own innovation record while social learners in chains inherited the record of someone else. Participants in groups could observe the innovation record of any other member of their group at any point during the session.

## Results

Initial analyses confirm that results from both chains and groups replicate well-established previous findings: the score of social learners increased both across generations within chains and with group size for groups. The analyses below control for accumulated time, to address the criticism that individual control conditions should provide individual learners with the same amount of accumulated time than social learners.

### Score Across Treatments When Controlling for Accumulated Time.

**Fig. 2.**
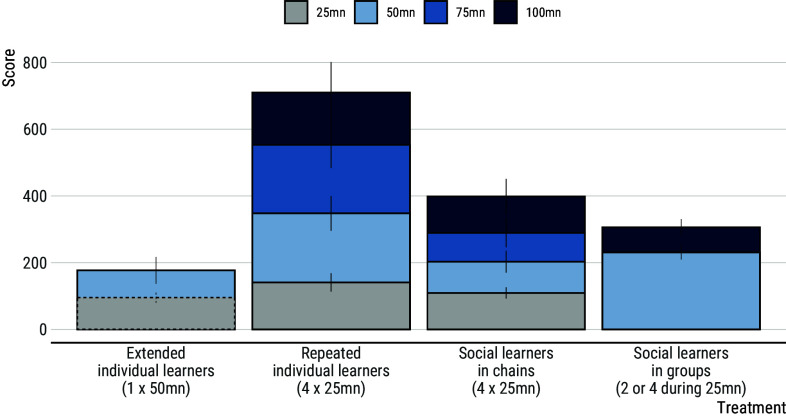
Score over time across the four experimental conditions. Gray bars show the score achieved by participants all of whom learned individually for 25 min. Scores do not differ between conditions until this point. Repeated individual learners who took part in multiple sessions reliably outperform participants from any other treatment after both 50 min (light blue bars) and 100 min (dark blue bars) of accumulated time. Experimental sessions were 25 min long in all treatments except for extended individual learners who took part in 50-min long sessions. The dashed line illustrates their performance after 25 min for comparison purposes. Error bars show s.e.m.

In our experiment, participants learned individually for 25 min in 3 treatments (Fig. 2). Analyses confirm that scores do not differ between treatments up to this point (extended versus repeated individual learners, 95% CI: (−61.7; 16.6), mean = −23.0; repeated individual learners versus learners in chains, 95% CI: (−26.4; 69.5), mean = 21.4). After 50 min of accumulated time, scores reached by extended individual learners, learners in chains, and learners in groups are comparable (e.g. extended individual learners versus learners in groups, 95% CI: (−91.7; −39.5), mean = −25.6). However, repeated individual learners reliably outperform learners in any other treatment (e.g. contrast with learners in groups, 95% CI: (35.6; 215.6), mean = 124.4). The same pattern is observed after 100 min of accumulated time, with learners in chains reaching comparable scores as learners in groups (generation 95% CI: (−45.7; 233.0), mean = 90.0), and repeated individual learners reliably outperforming learners from any other treatment (e.g. contrast with learners in chains 95% CI: (146.6; 423.4), mean = 285.1).

### Repeated Individual Learners Outperform Learners in Chains.

**Fig. 3.**
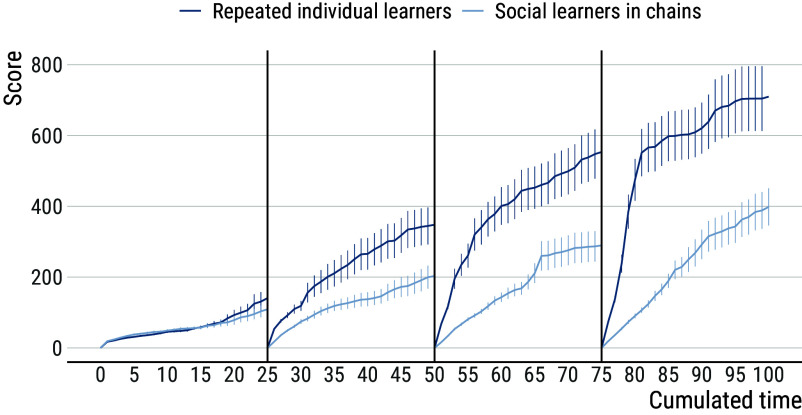
Score over time among repeated individual learners and social learners in chains. The score of individual and social learners follows a similar trend during the first 25-min session. However, repeated individual learners start outperforming social learners in chains from the second session onward. Vertical lines show the end/beginning of the 25-min sessions. Error bars show s.e.m.

To better understand these trends, we ran additional analyses investigating the various trade-offs that learners face in different experimental conditions. We start by further analyzing data from chains compared to repeated individual learners. As noted above, it is sometimes argued that the latter provides the most direct nonsocial control condition for the former. We found that repeated individual learners greatly outperform learners in chains. To understand why, we reconstructed learners’ scores over time, providing insights into how learners deal with the information received from the previous session (Fig. 3). Unsurprisingly, the scores of individual and social learners follow very similar trends during the first 25-min session. However, repeated individual learners start outperforming social learners in chains from the second session onward.

**Fig. 4.**
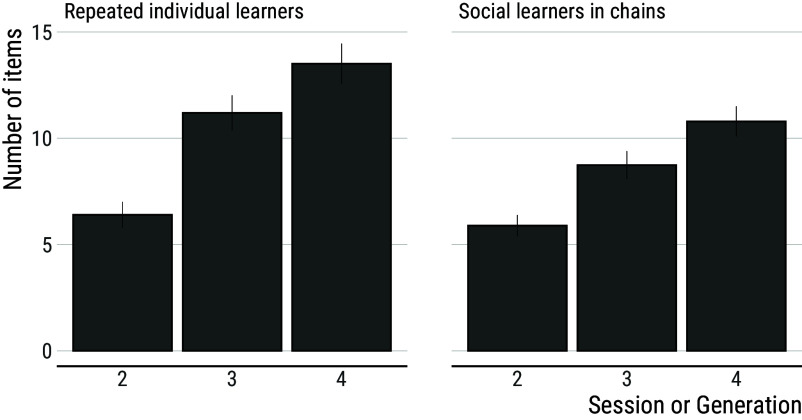
Number of items inherited by repeated individual learners and social learners in chains. Repeated individual learners inherit more items than social learners in chains, reflecting the better overall performance of the former. Error bars show s.e.m.

**Fig. 5.**
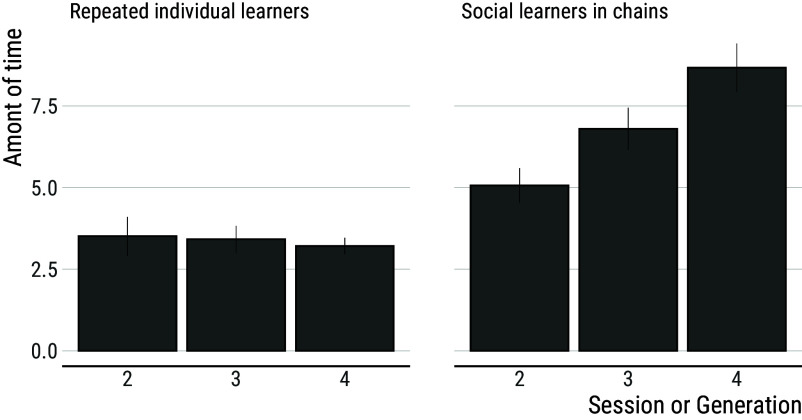
The time it takes participants to reproduce the inherited items increases across generations among social learners but not among repeated individual learners. Error bars show s.e.m.

Notably, it takes repeated individual learners less time than social learners in chains to reach a score comparable to the one reached at the end of the previous session. This is surprising given that, due to their higher scores, repeated individual learners inherit more items than social learners in chains, and more items should require more time to reconstruct (Fig. 4). Analyses of the number of items inherited per generation indicate that this number increases across generation among social learners (generation 95% CI: (1.81; 2.46), mean = 2.13) but does so faster among individual learners (generation × individual learning 95% CI: (0.40; 1.30), mean = 0.85). Yet, the time it takes participants to reproduce the inherited items increases across generations among social learners (generation 95% CI: (1.14; 2.06), mean = 1.61) but not among individual learners (generation 95% CI: (−0.67; 0.50), mean = −0.09; see Fig. 5).

This means that, even though they had more items to reproduce, repeated individual learners are faster than social learners in chains at reproducing what has been discovered before. After 3 sessions, repeated individual learners spend an average of 3.2 min reproducing inherited items leaving them 87% of the session to innovate further. In comparison, social learners spend an average of 8.7 min reproducing inherited items leaving them only 65% of the session duration to innovate further.

### Social Learners in Chains versus Social Learners in Groups.

Groups are an alternative way to organize social learners in cumulative culture experiments. Our results show that repeated individual learners greatly outperform social learners in groups when controlling for accumulated time (Fig. 2). This is likely to result, at least partly, from the benefits of the accumulated experience of individual learners documented above. However, additional effects may play a role in the respective performance of participants in groups versus chains. Hence we now compare groups with chains, allowing us to consider only learners who are naÃ¯ve at the beginning of their experimental session (Fig. 1).

**Fig. 6.**
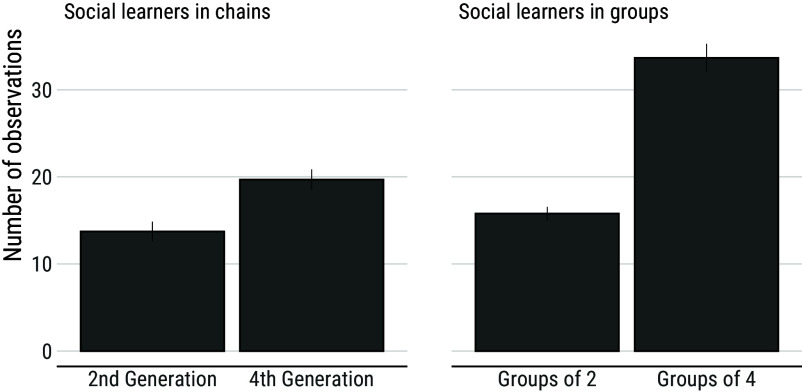
Number of monitoring events among social learners in chains and groups. The number of times participants attended to the record of another individual increases with both generation and group size. Comparisons between chains and groups indicate that this number is comparable between learners in groups-of-2 and second-generation learners in chains. However, it is reliably higher among learners in groups-of-4 compared to fourth-generation learners in chains. Error bars show s.e.m.

Results show that groups perform slightly better than chains after 50 min of accumulated time and slightly worse after 100 min of accumulated time, although none of these differences are reliable (Fig. 2). To further understand this pattern, we analyzed the cost of social learning in both treatments. Compared to social learners in chains who can focus on learning socially at the beginning of the experimental session, social learners in groups must alternate between bouts of individual and social learning. Consequently, we used the number of times participants attended to another participant’s record as a proxy for the cost of social learning. As shown above, using a different metric, participants who are later in a transmission chain inherit more items, meaning that they must attend to their demonstrator more often (Fig. 6). Analyses confirm this with fourth-generation social learners in chains observing their demonstrator more often than second-generation social learners in chains (contrast 95% CI: (0.91; 8.83), mean = 4.76). A similar effect is observed among social learners in groups who tend to have more opportunities to learn from others as group size increases (contrast 95% CI: (14.3; 21.2), mean = 17.7). However, compared to social learners in chains who attend to a single demonstrator whatever generation they belong to, social learners in groups must attend to more demonstrators as group size increases. Comparison between chains and groups indicates that the cost of monitoring demonstrators is comparable between second-generation learners in chains and learners in groups-of-2 (contrast 95% CI: (−2.44; 4.66), mean = 1.07), while it is reliably lower for fourth-generation learners in chains compared to learners in groups-of-4 (contrast 95% CI: (−18.2; −9.9), mean = −14.0). This means that, as the number of participants involved increases, the cost of social learning scales up faster in groups than in chains.

**Fig. 7.**
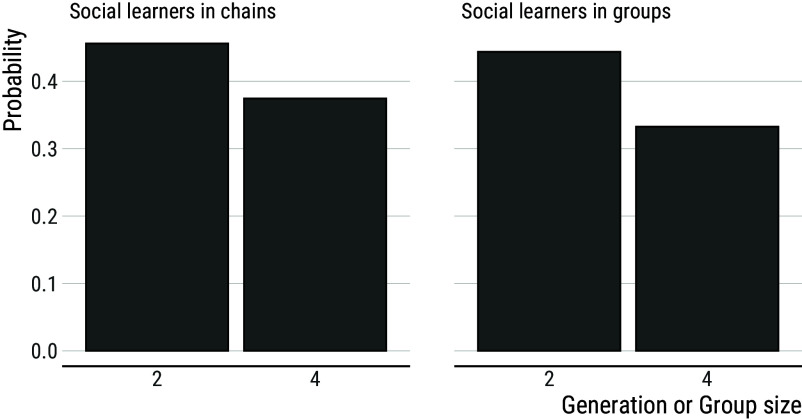
Probability of producing a combination that has not been produced before. Individuals’ mean probability of producing a combination that has not been produced before by any other member of their chain or group decreases with both generation and group size. Comparisons between chains and groups indicate that this probability is comparable between learners in groups-of-2 and second-generation learners in chains, but reliably lower among learners in groups-of-4 compared to fourth-generation learners in chains.

Another limitation of being part of a group is that group members will spend time solving the same problems, while learners in chains will tend to tackle novel problems. Hence participants in groups should produce more redundant work than participants in chains, especially as group size increases. To test this, we compared individuals’ average probability of producing a combination that has not been produced before by any other member of their chain or group (Fig. 7). Within groups, this probability reliably decreases from 0.45 for participants in groups-of-2 to 0.34 for participants in groups-of-4 (contrast 95% CI: (−0.12; −0.08), mean = −0.10). Comparisons between chains and groups indicate the probability of producing a combination that is novel is comparable between learners in groups-of-2 and second-generation learners in chains (0.45 and 0.46, respectively; contrast 95% CI: (−0.04; 0.01), mean = −0.02). However, this probability is reliably lower among learners in groups-of-4 compared to fourth-generation learners in chains (0.34 and 0.39, respectively; contrast 95% CI: (−0.07; −0.02), mean = −0.05).

### Individual Learners Perform Better When Learning Time is Distributed over Multiple Sessions.

Finally, we examine the difference between extended individual learners who took part in a single 50-min session and repeated individual learners who took part in two 25-min sessions. Results indicate that, although participants achieve comparable scores over the first 25 min, repeated individual learners reliably outperform extended individual learners after 50 min of accumulated time (Fig. 2). This is surprising because, in contrast to extended individual learners, repeated individual learners must recreate the items they inherited at the beginning of the second session. To investigate why, we analyzed the number of combinations produced by individuals over a comparable period of time as a proxy for effort. Over the first 25 min, extended individual learners produce a comparable number of combinations to repeated individual learners (estimated mean number of combinations = 209.9, s.d. = 8.9 and mean = 227.7, s.d. = 9.0, respectively; contrast 95% CI: (−4.58; 40.34), mean = 17.84). However, extended individual learners produce fewer combinations during the second 25 min of their experimental session compared to the first 25 min (mean = 176.3, s.d. = 9.9; contrast 95% CI: (−50.1; −17.6), mean = −33.6). Among repeated individual learners, no reliable difference is observed between the first and second sessions (mean = 227.7, s.d. = 9.0 and mean = 220.9, s.d. = 9.0, respectively; contrast 95% CI: (−21.9; 8.6), mean = −6.8). Additional contrasts confirm that extended individual learners produce fewer combinations during the second part of their experimental session compared to the second session of repeated individual learners (contrast 95% CI: (−68.8; −20.1), mean = −44.6).

## Discussion

Over more than two decades, experiments have been used to test various hypotheses concerning cultural evolution generated by a body of formal theory developed over the last 50 y. In this paper, we have reviewed key findings to have arisen from cultural evolution experiments, as well as outlined major methodological advances. We then turned to a specific outstanding issue, reflecting criticisms of previous experiments (63): what is the relevant individual learning control condition when attempting to demonstrate cumulative culture in the lab? We suggest, and demonstrate using an original experimental study involving commonly used experimental designs, that the answer to this question is not as straightforward as first appears. Rather than arguing over whether cumulative culture has or has not been demonstrated (an issue which partly depends on which definition a researcher uses, and may well be untestable in the lab in any case), we think that it is more constructive to consider the distinct challenges and trade-offs that social and individual learning entail.

In our experiment, the comparison between social learners in chains and repeated individual learners provides the most straightforward test for the proposed baseline condition. In both treatments, participants took part in sessions with equivalent duration for an equivalent number of generations/sessions. Perhaps surprisingly, we found that repeated individual learners greatly outperformed social learners in chains. Due to the improvement across generations in chains of social learners, a process-focused definition would likely categorize this as a clear case of cumulative culture. In contrast, the fact that performance is lower among social learners than individual learners would lead proponents of a product-focused definition to reject this conclusion. However, our results also indicate that the suitability of the implemented baseline condition is questionable from a methodological perspective. Our experiment highlights how the experience accumulated by individual learners over multiple sessions translates into greater efficiency at reproducing the items that compose their innovation record, and consequently higher scores. This is a consequence of a well-known relationship between individuals’ proficiency at a task and the amount of experience they have (a relationship commonly referred to as a “learning curve” (66)). By providing participants in individual control conditions with an equivalent amount of accumulated time as an entire chain, participants in individual control conditions end up having much more experience with the task than participants in chains.

Among individual learners, the effect of accumulated experience on proficiency is such that the amount of time it takes to reproduce their innovation record remains stable across sessions, even though the record increases in size. In comparison, social learners need an increasing amount of time to reproduce what has been discovered before, which substantially reduces the time they can devote to innovating further. Among social learners, the time needed to reproduce what has been discovered before goes from 20% of session duration after one generation to 35% after three generations. This illustrates the effect of variable acquisition costs, which result from the increasing amount of accumulated knowledge and skills that participants in later generations must acquire. Theoretical models have shown that, when acquisition costs increase over time, cumulative culture may come to a point where individuals spend all their time acquiring what previous generations have discovered before, which can stall the cumulative process (67).

The fact that acquisition costs affect social learners to a greater extent than individual learners shows that substantial amounts of cultural loss occur within chains, even in a setting where innovations are automatically transmitted across generations. This is because individuals’ performance not only depends on the accumulation of knowledge, but also skill (68). Furthermore, knowledge is never fully transmitted between individuals. For instance, individuals’ unsuccessful combinations are not shared across generations, even though they might help to navigate the problem space.

The proposed control condition might be even less suitable when the experimental design involves groups. In addition to the effects discussed above, groups will also produce more redundant work than single participants. This is because participants in groups work on the same problem at the same time. In our experiment, learners in groups are likely to discover a low-level innovation (e.g., a stone tool) faster than individual learners. Yet, every group member will have dedicated their time to solving this same problem. In comparison, most participants in chains do not need to spend time figuring out how to produce a stone tool (much like we do not need to spend time figuring out how to make fire from flint and pyrite). This results in more opportunity to innovate further along the chains, especially as the number of participants involved in the process scales up.

The comparison between extended and repeated individual learners also reveals that controlling for accumulated time generates another type of limitation linked to participants’ motivation. Indeed, we observed a drop in the number of combinations produced (a proxy for effort) among individual learners within a single 50-min-long session. In our experiment, the appropriate control for our social conditions required individual learners to take part in a 100-min-long experiment (split over multiple days for repeated individual learners). In other experiments, controlling for time would have required individual learners to take part in 640-min-long sessions (45). Beyond being impractical, sessions this long are likely to significantly decrease motivation and/or increase cognitive fatigue.

Importantly, the effects reported here likely do not apply equally to all experimental designs. In some experiments, social learning phases alternate with building phases, so social learning would not reduce building time as occurred in our transmission chain condition. Other experiments have implemented a phase during which social learners can select a single demonstrator from several options, e.g. based on the demonstrators’ score or reputation. This would attenuate the cost of monitoring multiple demonstrators as observed in our group condition. Features of the experimental task are also likely to determine how acute the reported effects are. Here, we used a task where participants had to discover increasingly complex and nested innovations. As shown above, this results in increasing acquisition costs over time, which may be a central feature of open-ended cumulative culture (69). Other tasks, however, might involve solutions of variable efficiency but equivalent learning difficulty (43, 61). In our experiment, the effect of variable acquisition costs might also be reduced by providing learners with tools instead of recipes. This would make social learners immediately ready to innovate further and would reduce the gap with individual learners, although individual learners would still have more accumulated experience overall.

Even though the reported effects are likely to be mediated by experimental designs and tasks, we believe that controlling for accumulated time is not a solution as straightforward as it appears. Rather we suggest acknowledging that the way humans solve problems individually often differs qualitatively from the way we solve them collectively. Individually, problem-solving abilities are strongly limited by how much information individuals can process and how much time they can dedicate to it. Collectively, limitations in problem-solving abilities will mostly result from our ability to coordinate and transmit information and skills. What solving tasks collectively does is precisely circumvent the bottlenecks limiting the achievements of isolated individuals, such as their limited lifespan.

In conclusion, we have presented some original data that examines the various trade-offs inherent in different experimental designs and control conditions commonly used in the experimental study of cultural evolution. We hope that this contributes to clarifying previous findings related specifically to cumulative culture, but also to broader considerations in the design of cultural evolution experiments. A well-developed and continually evolving set of experimental designs and methods promises to be a powerful tool within a broad science of cultural evolution, as initiated 50 y ago.

## Materials and Methods

### Participants.

570 undergraduate participants (342 women) at the University of Wisconsin-Madison completed the experiment in exchange for course credit. Participants ranged in age from 18 to 43 y (mean 18.8 y, s.d. 1.5 y). Students received credit based on the duration of each completed session (25 min or 50 min). Participants assigned to the repeated individual learning condition who completed all four sessions were awarded additional credit as an incentive to prevent dropout. Informed consent was obtained from all subjects before starting the experiment. Ethical approval was given by the University of Wisconsin-Madison. Six participants from the repeated individual learning condition did not complete all four sessions and were excluded from all statistical analyses (4 completed 2 sessions and 2 completed 3 sessions).

### Task.

Participants played a computer game programmed in Object Pascal with Delphi 6 that simulated a real-world innovation process in which the production of complex artifacts depended on the discovery of high-level innovations (62). Discovering these innovations was contingent on the discovery of lower-level innovations. Both low- and high-level innovations resulted from a specific production process that was initially unknown to participants. Players were initially provided with six basic resources that could be used without limit and combined using a workshop panel containing four slots. After dropping between one and four resources into this panel, players could trigger an automatic refining process at no cost and without any limit by clicking on a “Try” button. Innovations arose when players produced a combination that belonged to a list of predetermined successful combinations. A specific slot displayed the result: a red cross when the combination was unsuccessful, a new item otherwise. When discovered, new items could be in turn associated with other items to produce higher-level innovations. All combinations were allowed, including those involving the repeated use of the same item. The order of the items in the workshop panel had no effect on the result, so that 209 unique combinations could be produced from six initial resources. The production of new items led to a combinatorial explosion, so that 1,000 different combinations could be produced after the discovery of four new items/innovations. In total, 27 additional items (all useful) could be generated from the six initial resources. The accumulation of innovations could result in the production of complex tools (such as axes) that potentially allowed players to get logs by cutting trees. Basic logs required at least eight innovations to be produced and were the minimal element that could be dropped into a three-slot totem pole panel, which provided players with a totem score. Logs could be refined when combined with relevant tools (such as carving tools, pigments, brushes and so on) in the workshop panel. 115 different logs could be produced, so that a total of 142 innovations and 266,915 unique totems could be generated. Once a tool was discovered, the recipe for its creation, i.e. the list of items that must be combined to create the tool, was displayed in an “innovation record.” Participants could review their own past discoveries by clicking on a tool to see its recipe.

### Score Calculation.

Each of the 115 different logs was associated with a unique value that was randomly attributed within a range of scores that depended on the log’s complexity. The complexity of logs was defined by the number of innovations that was required to produce them. This means that logs with more underlying innovations were always more rewarding, although two logs with the same number of underlying innovations didn’t have the same value. The score of a totem, which depended on the value of the logs and their diversity, was calculated as follows:

$$Score_{\text{Totem}}=\left(1+0.15\alpha \right)\left(Score_{\text{Log1}}+Score_{\text{Log2}}+Score_{\text{Log3}}\right)$$

With *α* taking the value 0, 1, or 2 depending on whether the totem pole involved 1, 2, or 3 different logs. Totem scores ranged from 50 to 7,410 points. The players’ final score equaled to the score of the best totem they built plus 15 points for each new items they produced.

### Tutorial and Pregame Information.

Before starting, the players completed a tutorial during which basic actions, such as dragging and dropping resources in the workshop panel, had to be completed. Players were informed that the ultimate aim of the game was to build a totem pole, that innovations have to be produced before being able to produce logs and that these logs could be used and refined to make totems. They were also informed that their score depended on the number of new items they will produce and the value of their totem. The fitness function that determined the value of a totem was unknown by the players.

### Treatments.

Participants were randomly assigned to one of four experimental designs as specified in the main text. All participants had access to an information panel whose content varied according to the treatment. Extended individual learners were provided with their own score and a record of their own innovations (alongside their best totem if any). Participants from other treatments benefited from additional information and could switch between their own record and others’ record by clicking on an anonymized name (e.g. “Participant 2”) and associated score. Repeated individual learners and social learners in chains experienced the exact same conditions, each starting a new session by inheriting the innovation record produced during the previous session. The only difference is that repeated individual learners inherited their own innovation record while social learners in chains inherited the record that someone else had produced. Participants in groups could observe the innovation record of any other member of their group at any point during the session (participants’ score was updated every 10 s). Innovation records provided the recipes for creating the tools, but not the tools themselves. To use these tools in further combinations, the tools, including all of the constituent parts, had first to be reproduced by the participant observing the innovation record. Participants were free to decide whether they wanted to observe an innovation record and when they wanted to do so. Innovation records could be observed without any limitations in all treatments.

### Statistical Analysis.

We ran a series of Bayesian models in R (70). Models were fitted using the rethinking package (71) and 95% credible intervals were used to make inferences. Three models were run to compare the score of participants after 25, 50, and 100 min of accumulated time. For the 25-min model, scores reached after 25 min by extended individual learners, at the end of the first session by repeated individual learners, and at the end of the first generation by social learners in chains were considered. We fitted a linear model with “Score” as the outcome variable, and two dummy variables (one for repeated individual learners and one for social learners in chains) as predictors. For the 50-min model, scores reached after 50 min by extended individual learners, at the end of the second session by repeated individual learners, at the end of the second generation by social learners in chains, and at the end of their session by social learners in groups-of-2 were considered. We fitted a linear model with “Score” as the outcome variable, three dummy variables (one for repeated individual learners, one for social learners in chains, one for social learners in groups) as predictors, and “Group identity” as a random effect for social learners in groups. For the 100-min model, scores reached at the end of the fourth session by repeated individual learners, at the end of the fourth generation by social learners in chains, and at the end of their session by social learners in groups-of-4 were considered. We fitted a linear model with “Score” as the outcome variable, and two dummy variables (one for social learners in chains and one for social learners in groups) as predictors, and “Group identity” as a random effect for social learners in groups.

For the number of items inherited among repeated individual learners and social learners in chains, we fitted a linear model with “Number of items inherited” as the outcome variable, “Generation,” one dummy variable for repeated individual learners and their interaction as predictors, and “Participant (or Chain) identity” as a random effect.

For the time needed to reproduce inherited items among repeated individual learners and social learners in chains, the time needed to reproduce inherited items was attributed to participants based on the inherited item that they reproduced at the latest time. We fitted a linear model with “Time” as the outcome variable, “Generation,” one dummy variable for repeated individual learners and their interaction as predictors, and “Participant (or Chain) identity” as a random effect. Note that not all participants reproduced the entire set of inherited items (2 individual learners and 1 social learner at session/generation 2; 2 social learners at generation 3; and 6 social learners at generation 4). All observed the inherited innovation record except one of the individual learners.

For the cost of monitoring among social learners in chains and groups, we used the number of times individuals switched between their own record and others’ record as a proxy for the cost of monitoring cultural models. This number was calculated for second- and fourth-generation social learners in chains and social learners in groups of two and four. We fitted a linear model with “Number of observations” as the outcome variable, and three dummy variables (one for fourth-generation social learners in chains, one for social learners in groups-of-2, one for social learners in groups-of-4) as predictors.

For the probability of producing redundant work among social learners in chains and groups, for each participant, we computed both how many of their combinations had not been produced before by any other member of their chain or group and their total number of combinations produced. We ran two different models. In the first, we analyzed data from social learners in groups-of-2 and first- and second-generation social learners in chains. We fitted a binomial regression with “Number of novel combinations” as the response variable, one dummy variable for social learners in chains as the predictor, and “Group (or Chain) identity” as a random effect. In the second, we analyzed data from social learners in groups-of-2, social learners in groups-of-4 and first-, second-, third-, and fourth-generation social learners in chains. We fitted a binomial regression with “Number of novel combinations” as the response variable, two dummy variables (one for social learners in groups-of-4 and one for social learners in chains) as predictors, and “Group (or Chain) identity” as a random effect.

For the number of combinations produced among extended and repeated individual learners, we used the number of combinations produced by extended individual learners after 25 and 50 min, and the number of combinations produced by repeated individual learners at the end of their first and second sessions were considered. We fitted a linear model with “Number of combinations” as the outcome variable, three dummy variables (one for extended individual learners after 50 min, one for repeated individual learners after first session, and one for repeated individual learners after the second session) as predictors, and “participant identity” as a random effect.

## Data Availability

Participant responses in the experiment data have been deposited in Open Science Framework (OSF) (https://osf.io/th2zc/) (72).
